# Therapist effects on client drinking across four motivational interviewing sessions: a longitudinal analysis of process predictors

**DOI:** 10.1186/1940-0640-7-S1-A89

**Published:** 2012-10-09

**Authors:** Molly Magill, Robert Stout, Timothy Apodaca

**Affiliations:** 1 Center for Alcohol and Addiction Studies, Brown University, Providence, RI, USA; 2 Decisions Sciences Institute, Pacific Institute for Research and Evaluation, Providence, RI, USA

## 

Scientific attention has shifted to understanding the underlying mechanisms that account for the efficacy of brief intervention (BI) based on the principles of motivational interviewing (MI). A large portion of work thus far has emphasized therapist skills and client language mechanisms in the context of single-session interventions. The present study examined three global therapist variables of clinical importance to MI (therapist focus on client ambivalence/discrepancy, therapist emphasis on client commitment to change, and therapist assessment of client goals/drinking) in the context of a multi-session intervention. The sample included adults with alcohol use disorders participating in the multi-site Matching Alcoholism Treatments to Client Heterogeneity (MATCH) clinical trial. Binomial generalized estimating equations examined therapist intervention effects on alcohol use over a 12-week treatment period (four sessions). The main effects analysis showed that therapist emphasis on commitment had a positive effect on drinking reduction for aftercare patients (odds ratio [OR], .64; p < 0.001). However, the opposite pattern was found for therapist focus on client ambivalence/discrepancy (aftercare OR, 1.31; p < 0.001) (outpatient OR, 1.10; p = 0.018) and assessing goals/drinking (aftercare OR, 1.22; p = 0.043) (outpatient OR, 1.27, p < 0.001) in both samples. Therapist-reported intervention foci are important to subsequent patterns of drinking within a multi-session MI intervention. The unexpected negative effect of focus on client ambivalence and assessment of goals and drinking suggests that therapists may use these interventions in reaction to client alcohol use, but are unsuccessful in movement toward resolution. Patterns of alcohol use within treatment moderately predicted follow-up outcome, supporting the importance of these clinical processes.

